# Vaginal Reconstruction in Patients with vaginal agenesis: Options and Outcome: A single-center experience

**DOI:** 10.12669/pjms.39.1.6356

**Published:** 2023

**Authors:** Saida Abrar, Raheela Mohsin, Zia-Ul-Islam

**Affiliations:** 1 Dr. Saida Abrar, Fellowship Urogynecology, Aga Khan University, Pakistan. Assistant Professor OBGYN, Lady Reading Hospital, Peshawar, Pakistan; 2 Dr. Raheela Mohsin Rizvi, Clinical Fellowship Urogynecology Sydney, Australia. Professor and Director, OBGYN, OMI Hospital, Karachi, Pakistan; 3 Dr. Mohammed Zia-Ul-Islam, Consultant Plastic Surgeon, Aga Khan University Hospital, Karachi, Pakistan

**Keywords:** Mayer-Rokitansky-Kuster-Hauser (MRKH) syndrome, Androgen insensitivity syndrome (AIS), Vaginal agenesis, Vaginal reconstruction

## Abstract

**Objectives:**

To assess the postoperative functional, anatomical outcome and complications of various surgical procedures of vaginoplasty performed for patients with vaginal agenesis at our institution.

**Methods:**

This was a cross-sectional study of 14 patients (age range 17-40 years), who underwent vaginoplasty at the Aga Khan University Hospital, Karachi, Pakistan between January 2008 to December 2018. We aimed to assess the anatomical outcomes in terms of vaginal depth, axis and functional outcome as painless and satisfactory vaginal intercourse.

**Results:**

The mean age and mean body mass index (BMI) of the cases were 26.8 ± 8.1 years and 27.7143 ± 4.6 respectively. All were phenotypically female, with only two cases of XY genotype. Two patients were married on presentation. On evaluation, four cases had Mayer-Rokitansky-Kuster-Hauser (MRKH) syndrome, three had androgen insensitivity syndrome (AIS), one case had congenital adrenal hyperplasia and six cases did not fit into any diagnosis. Associated renal anomalies were diagnosed in 14.3% of cases. The performed procedures were; Singaporean flap vaginoplasty (in four patients), Lee’s, modified McIndoe and pull-through vaginoplasty (in three each patients), and Davydov vaginoplasty (in one patient). One patient was complicated by intraoperative bladder injury (p<0.63) and two cases by vaginal stenosis (p<0.43). The mean operative time was 120 minutes and the mean estimated blood loss was 200mls. Postoperatively, the vaginal length varied from 6-10 cm with a normal vaginal axis and satisfactory sexual activity.

**Conclusions:**

Vaginal agenesis is associated with several sexual disorders and despite the various surgical options available, the best procedure in terms of fewer complications and best surgical outcome is yet to be determined.

## INTRODUCTION

Vaginal agenesis/ hypoplasia is defined as a complete absence of or a rudimentary vagina. It occurs in a variety of disorders, the most common being congenital conditions like MRKH and AIS with a global incidence of 1 in 4,000 and 1 in 13,000 respectively.^1^-^3^

MRKH syndrome is characterized by having a normal female phenotype and karotype, normal ovarian function but agenesis of the uterus and upper two-thirds of the vagina. On examination, they have normal secondary sexual characteristics, but the vagina is either completely absent or only 3-5 cm in length. It is usually associated with other congenital anomalies, the most common being urological, reported in 15-34% of cases.^4^ Class-I Mullerian anomaly is a rare type of MRKH, which is characterized by the development of the uterus, cervix, and upper part of the vagina but the absent lower part of the vagina.^4^

AIS is characterized by a phenotypically female appearance with XY genotype.^5^ It can be X-linked recessive or sporadic. Those with complete AIS present as females with an XXY genotype. These patients are usually diagnosed in adolescents when they present with primary amenorrhea, failure of the consummation of marriage, or more rarely primary infertility.

Herlyn–Werner–Wunderlich syndrome or OHVIRA syndrome is a complex urogenital anomaly of Mullerian and mesonephric duct, presenting as obstructed Hemi-vagina and ipsilateral renal agenesis/anomaly and uterus didelphys.^6^

Various surgical methods are described in the literature for the creation of neovagina. The various surgical methods include, McIndoe Reed technique, intestinal vaginoplasty (Baldwin), peritoneal vaginoplasty (Davydov), (Williams) vulvavaginoplasty, pudendal flap vaginoplasty, pull through, and Lees vaginoplasty.^4^,^7^ Surgical success is usually reported as successful coitus, or satisfaction evaluated by using validated questionnaires.^8^ Despite the various methods described, only limited studies address the best surgical method and most of them consist of cross-sectional, single-center studies with poor standardization and short follow-up. Hence, the best surgical method still remains controversial.^9^

This study aimed to describe the practice of vaginoplasty at our institution in patients with different sexual disorders presenting with vaginal agenesis. The primary objective was to find the postoperative functional, and anatomical outcome while the secondary objective was to find the postoperative complications, using data from medical chart reviews.

## METHODS

We conducted a retrospective cross-sectional study of 14 patients at Aga Khan University Hospital, Karachi who underwent vaginoplasty for vaginal atresia due to different disorders over 10 years (January 2008 to December 2018). Patient were identified by using the International Classification of Diseases, Ninth Revision code 70.79 for vaginoplasty.

Charts were reviewed and a structured performa was used for the data collection on demographic, clinical examination, operative notes, any complications, and postoperative follow-up. Women lost to follow-up at six months postoperatively, were excluded. All the surgeries were performed after written informed consent. The surgical team included mainly urogynecologist, with plastic surgeons only for the Singaporean vaginoplasty.

All patients were operated on under general anesthesia. The patient remained catheterized for 48 hours and received IV antibiotics, i.e. Ciprofloxacillin and Metronidazole for 48 hours postoperatively, followed by oral antibiotics for a week. Mold was kept for 24 hours in patients undergoing McIndoe’s technique, Davydov and Lee’s vaginoplasty only. Patients were then trained on daily self-dilatation with a hegar’s dilator for up to two months. The progress was assessed after one, two, and four weeks in the clinic. Sexual activity was allowed once healing was complete. Those who were sexually inactive were advised to continue self-dilation at least four times a week for up to six months.

### Ethical Approval:

The study was performed after exemption from the ERC for this study (ERC 2020-3552-10501).

## RESULTS

Only two of the 14 patients were married at the presentation. The conditions associated with vaginal agenesis were; MRKH syndrome-four cases, AIS-three cases, and congenital adrenal hyperplasia (CAH)- one case. Six cases did not fit into any specific diagnosis, with three cases having isolated distal vaginal atresia, one patient with an intact uterus but absent cervix and vagina while two patients had a variant of Herlyn–Werner–Wunderlich syndrome with uterus didelphys, ipsilateral renal agenesis/anomaly but absent distal vagina (Table-I).

**Table-I:** Demographic characteristics of the patient (n=14).

Variable	Mean ± SD or n (%)
Age (Mean ± SD)	26.86 ± 8.189
BMI (Mean ± SD)	27.7143 ± 4.66457
***Phenotype n (%)***	
Female	14 (100%)
Male	0
***Genotype n (%)***	
46-XX	12 (85.7%)
46-XY	2 (14.3%)
***Marital status n (%)***	
Married	2 (14.3%)
Unmarried	12 (85.7%)
***Diagnosis n (%)***	
MRKH	4 (28.57%)
AIS	3 (21.42%)
CAH	1 (7.14%)
Other	6 (42.85%)

Data are mean±standard deviation or number (%); MRKHMayer- Rokitansky-Kuster-Hauser, AIS- androgen insensitivity syndrome, CAH-congenital adrenal hyperplasia.

Seven patients presented with complaints of primary amenorrhea, three with failure of the consummation of marriage and four patients complained of lower abdominal pain. All patients had a normal female phenotype. Secondary sexual characteristics were normal in patients with MRKH syndrome, CAH, and isolated distal vaginal atresia while AIS cases had normal female breast but scanty pubic and axillary hair. Only two patients (14.3%) had associated renal anomalies. Preoperative vaginal length was 0-2cm (median length 1.75 with IQR 1 cm). Hormonal profile was in the normal female range except in AIS, who had elevated testosterone levels, and one patient with CAH, who had elevated 17 OH-progesterone.

Four patients underwent Singaporean flap vaginoplasty, one had a Laparoscopic Devidov procedure, while Lee’s, Pull through and McIndoe Reed vaginoplasty was performed in three patients each (Table-II).

**Table-II:** Operative Details of the patients (n=14).

Diagnosis (n=14)	Surgeries
MRKH (n=4)	Lee vaginoplasty (n= 3) Singaporean flap vaginoplasty (n=1)
AIS (n=3)	Singaporean flap vaginoplasty
Others (n=6) Isolated distal vaginal atresia (n=3) Intact uterus, absent cervix, and vagina (n=1) Uterus didelphys, two cervices with absent distal vagina (n=2)	Pull through vaginoplasty (n=2) McIndoe Reed vaginoplasty (n=3) Laparoscopic Devidov (n=1)
CAH (n=1)	Pull through vaginoplasty

Data are mean ± standard deviation or number (%); MRKHMayer- Rokitansky-Kuster-Hauser, AIS- androgen insensitivity syndrome, CAH-congenital adrenal hyperplasia.

**Table III:** Post-operative Anatomical Outcome and Complications.

Variables	Singaporean flap vaginoplasty	Pull through vaginoplasty	McIndoe Reed vaginoplasty	Laparoscopic Davidov vaginoplasty	Lee’s vaginoplasty	P-value
***Vaginal length (cm) Median (range)***						
After surgery						
At last follow-up	10 (0)	10 (1)	10 (1)	10 (0)	10 (0)	0.06
	8 (1)	6 (1)	6 (1)	6 (0)	7 (1)	
***Intraoperative bladder injury n (%)***	0	0	1	0	0	0.63
***Postoperative complications***						
Flap prolapse						
Shrinkage/stenosis	1	0	0	0	0	0.26
Bleeding	0	0	1	0	1	0.43
	0	0	1	0	0	0.99
***Functional success***						
Consummation of sexual activity						
Need of dilators	4	3	2	0	0	0.023
	0	0	1	0	1	0.308

Data are median (range) or number (%).

There was one case of bladder injury intraoperatively, during Lee’s vaginoplasty. It was timely identified and repaired. Operative time was 90-180 minutes with a mean operative time of 120 minutes and mean estimated blood loss of 200ml. Vaginal stenosis was the most common reported postoperative complication occurring in two patients, about six months postoperatively, one after the McIndoe technique, and one post-Lee’s vaginoplasty. Both these patients were not compliant with postoperative dilation. They were examined under anesthesia and dilation was done postoperatively until six weeks and a vaginal depth of 7cm was successfully achieved, six months postoperatively. Among patients with pudendal flap vaginoplasty, one patient was complicated with vaginal prolapse while two had hypertrophied scar at the thighs donor site. The postoperative vaginal length varied from 6-10cm, with a normal vaginal axis in all patients. All the patients reported satisfactory sexual activity in both partners on consummation of intercourse. The postoperative mean follow-up period was 24 months (range; 12-48 months). There was no need for second vaginal surgery in any of our patients.

## DISCUSSION

Vaginal hypoplasia is a rare anomaly with a significant psychological impact on a patient’s quality of life. A variety of disorders are implicated in their etiology. Correct diagnosis, evaluation of associated congenital anomalies, and psychosocial counseling before embarking on any intervention are pivotal steps of effective management. Surgery should be planned once the patient is mature enough and wishes for surgical correction.^10^

Despite the wide range of options available,^11^,^12^ the best surgical option in terms of the outcome, and sexual satisfaction, is yet to be found.^13^ A comparative study on 20 women’s with vaginal agenesis, subjected to either vaginal dilation or surgical vaginoplasty, found no significant difference in subjective satisfaction, anatomical outcome (p=0.09), and sexual functions (p=0.07). The suggested vaginal dilation could be considered as first management option for these cases.^14^ Thus, it is an effective method capable of achieving a vaginal length of about >6 cm in 91% of cases, but requires a long duration of treatment, self-motivation, and causes discomfort.^15^

Among the surgical methods, the McIndoe Reed procedure is one of the most frequently performed procedures. It involves dissection between the rectum and the bladder and placing a mold into the space created, usually covered by a split-thickness skin graft. Studies report 71-90% sexual satisfaction rates using this procedure.^10^,^16^

Davydov involves dissection of the rectovesical space, mobilization of a segment of the peritoneum abdominally, and its attachment to the introitus.^17^ The reported complications include injury to the bladder, rectum, and/ or ureter, peritonitis, vesicovaginal fistula formation, and lower urinary tract symptoms.^18^,^19^

Lee’s vaginoplasty is a new method associated with good outcomes, minimal morbidity, and satisfactory intercourse.^7^ It involves the creation of neovagina by using the serosal layer of the uterus and peritoneum for lining the neovagina via a combined laparoscopic and vaginal approach. The peritoneum can provide lubrication for intercourse while the uterine serosa provides additional strength to the tissue.^20^

Pudendal flap vaginoplasty involves dissection of the space for the neovagina, elevation of the flaps on the adductor muscle, passing them beneath the labial folds, and suturing together to create a tubular vagina followed by suturing the base of the flap to the medial side of labia majora bilaterally.^21^ Although many modifications have been introduced, overall it is associated with encouraging results, good aesthetic outcomes and no major complications.^22^ The reported postoperative complications include donor site scar hypertrophy and flap prolapse.

Pull-through vaginoplasty is done by dissection of the vaginal space until the palpable vaginal bulge is reached, the hematocolpos is drained, and the vaginal mucosa is attached to the perineum. Postoperative complications include vaginal stenosis, bladder or bowel injury, fistula formation, and infection.^23^

### Strength:

The strength of the study is that a variety of disorders causing vaginal agenesis and different types of surgical procedures were carried out for these conditions were addressed. Furthermore, all the procedures were carried out by a single surgeon.

### Limitations:

It include its retrospective study design and a small number of patients. However, disorders leading to vaginal agenesis are rare, and not many gynecologists are trained in these procedures, hence limited centers perform these surgeries.

## CONCLUSION

Despite various methods of vaginoplasty available for vaginal agenesis, it is difficult to suggest the best method of vaginoplasty based on this study due to its small sample size, however, our experience showed that Singaporean flap vaginoplasty had the best postoperative outcome in AIS due to the broad perineum and Lees vaginoplasty in MRKH syndrome. Future multi-institutional, long-term prospective studies are required to find the best surgical method suited to individual diagnosis.

### Authors’ Contribution:

**SA:** Conceived, designed, data collection, and manuscript writing.

**RMR:** Did the editing and final approval of the manuscript. Responsible for integrity and accuracy of the work.

**ZM:** Did the statistical analysis.

## References

[1] Akhtar AZ (1986). Congenital abnormalities of genital tract-vaginal defects. J Pak Med Assoc.

[2] Beksac MS, Salman MC, Dogan NU (2011). A New Technique for Surgical Treatment of Vaginal Agenesis Using Combined Abdominal-perineal Approach. Case Rep Med 2011.

[3] Herlin M, Bjorn AM, Rasmussen M, Trolle B, Petersen MB (2016). Prevalence and patient characteristics of Mayer-Rokitansky-Kuster-Hauser syndrome:a nationwide registry-based study. Hum Reprod.

[4] Passos IMPE, Britto RL (2020). Diagnosis and treatment of mullerian malformations. Taiwan J Obstet Gynecol.

[5] Gulia C, Baldassarra S, Zangari A, Briganti V, Gigli S, Gaffi M (2018). Androgen insensitivity syndrome. Eur Rev Med Pharmacol Sci.

[6] Gunduz R, Agacayak E, Evsen MS (2021). OHVIRA syndrome presenting with acute abdomen findings treated with minimally invasive method:three case reports. Acta Chir Belg.

[7] Lee JM, Huang CY, Wu KY, Yen CF, Chern B, Lee CL (2014). Novel technique of neovagina creation with uterine serosa in the treatment of vaginal agenesis associated with mullerian agenesis. Gynecol Minimal Invas Therapy.

[8] Cheikhelard A, Bidet M, Baptiste A, Viaud M, Fagot C, Khen-Dunlop N (2018). MRKH Study Group Surgery is not superior to dilation for the management of vaginal agenesis in Mayer-Rokitansky-Küster-Hauser syndrome:a multicenter comparative observational study in 131 patients. Am J Obstet Gynecol.

[9] Callens N, De Cuypere G, De Sutter P, Monstrey S, Weyers S, Hoebeke P (2014). An update on surgical and non-surgical treatments for vaginal hypoplasia. Hum Reprod Updat.

[10] Gari A (2017). Mclndoe Neovagina in patients with Mullerian Agenesis:A single center experience. Pak J Med Sci.

[11] Paul KG, Kosuri KC, Bayad H (2020). Congenital uterovaginal abnormalities, it's embryogenesis, surgical management and clinical implications. Obstet Gynecol Sci.

[12] Zafar M, Saeed S, Kant B, Murtaza B, Dar MF, Khan NA (2007). Use of amnion in vaginoplasty for vaginal atresia. J Coll Physicians Surg Pak.

[13] Laufer MR (2002). Congenital absence of the vagina:in search of the perfect solution When, and by what technique, should a vagina be created. Curr Opin Obstet Gynecol.

[14] Apfel VR, Takano CC, Marquini GV, Jarmy di Bella de ZIK, Girao MJBC (2022). Treatment for vaginal agenesis:A prospective and comparative study between vaginal dilation and surgical neovaginoplasty. Int J Gynaecol Obstet.

[15] Wee A, Bagchi T, Kimble R (2022). Creation and maintenance of neo-vagina with the use of vaginal dilators as first line treatment:Results from a quaternary paediatric and adolescent gynecology service in Australia. Aust N Z J Obstet Gynaecol.

[16] Karapınar OS, Ozkan M, Okyay AG, Sahin H, Dolapcıoglu KS (2016). Evaluation of vaginal agenesis treated with the modified McIndoe technique:A retrospective study. J Turk Ger Gynecol Assoc.

[17] Davydov S (1969). Colpopoiesis from the peritoneum of the ueterorectal space. Obstet Gynecol.

[18] Giannesi A, Marchiole P, Benchaib M, Chevret-Measson M, Mathevet P, Dargent D (2005). Sexuality after laparoscopic Davydov in patients affected by congenital complete vaginal agenesis associated with uterine agenesis or hypoplasia. Hum Reprod.

[19] Baruch Y, Nale R, Parma M, Fatta Di S, Fedele L, Candiani M (2020). Lower urinary tract symptoms in patients with Mayer-Rokitansky-Kuster-Hauser syndrome after neo-vagina creation by Davydov's procedure. Int Urogynecol J.

[20] Dargent D, Marchiol!e P, Giannesi A, Benchaïb M, Chevret-M“easson M, Mathevet P (2004). Laparoscopic Davydov or laparoscopic transposition of the peritoneal colpopoeisis described by Davydov for the treatment of congenital vaginal agenesis:The technique and its evolution. Gynecol Obstet Fertil.

[21] Fujioka M, Hayashida K, Murakami C (2014). Vulvar reconstruction should be performed using gluteal-fold perforator flap because of less morbidities and complications. Revista do Colégio Brasileiro de Cirurgioes.

[22] Fujiwara M, Suzuki T, Ohta Y, Okada E, Fukamizu H, Tokura Y (2019). Elevation of Thin Pudendal Artery Flap Using Fat Thickness Data in Vulvovaginal Reconstruction. Indian J Surg.

[23] Mansouri R, Dietrich JE (2015). Postoperative course and complications after pull-through vaginoplasty for distal vaginal atresia. J Pediat Adolesc Gynecol.

